# Quantitative Analysis of the Cerebral Vasculature on Magnetic Resonance Angiography

**DOI:** 10.1038/s41598-020-67225-w

**Published:** 2020-06-23

**Authors:** Pulak Goswami, Mia K. Markey, Steven J. Warach, Adrienne N. Dula

**Affiliations:** 1 Department of Neurology, Dell Medical School at The University of Texas, Austin, Texas USA; 2 Department of Biomedical Engineering, University of Texas, Austin, Texas USA; 3 Department of Oncology, Dell Medical School at The University of Texas, Austin, Texas USA; 4 Department of Imaging Physics, The University of Texas MD Anderson Cancer Center, Houston, Texas USA; 5 Department of Diagnostic Medicine, Dell Medical School at The University of Texas, Austin, Texas USA

**Keywords:** Stroke, Diagnostic markers, Translational research, Computational science, Biomedical engineering

## Abstract

The arterial connections in the Circle of Willis are a central source of collateral blood flow and play an important role in pathologies such as stroke and mental illness. Analysis of the Circle of Willis and its variants can shed light on optimal methods of diagnosis, treatment planning, surgery, and quantification of outcomes. We developed an automated, standardized, objective, and high-throughput approach for categorizing and quantifying the Circle of Willis vascular anatomy using magnetic resonance angiography images. This automated algorithm for processing of MRA images isolates and automatically identifies key features of the cerebral vasculature such as branching of the internal intracranial internal carotid artery and the basilar artery. Subsequently, physical features of the segments of the anterior cerebral artery were acquired on a sample and intra-patient comparisons were made. We demonstrate the feasibility of using our approach to automatically classify important structures of the Circle of Willis and extract biomarkers from cerebrovasculature. Automated image analysis can provide clinically-relevant vascular features such as aplastic arteries, stenosis, aneurysms, and vessel caliper for endovascular procedures. The developed algorithm could facilitate clinical studies by supporting high-throughput automated analysis of the cerebral vasculature.

## Introduction

**Figure 1:**
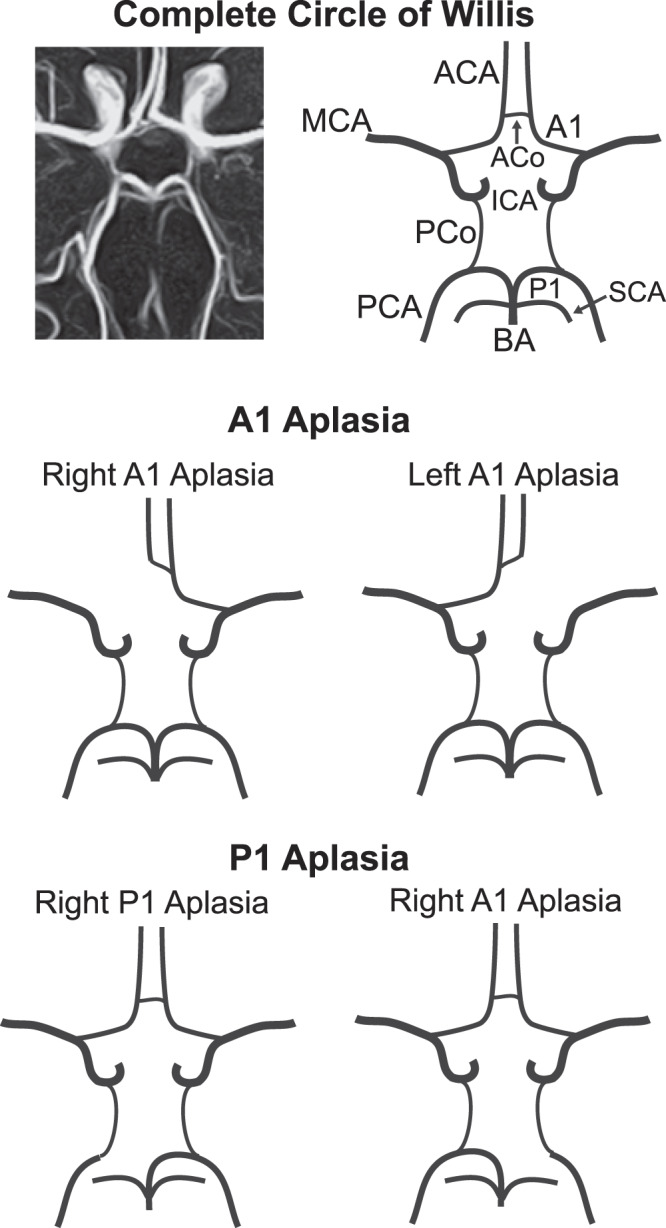
Anatomy of the complete Circle of Willis (top) along with the A1 aplasia (middle) and P1 aplasia (bottom) variant structures. MCA: middle cerebral artery, ACA: anterior cerebral artery, ACo: anterior communicating artery, A1: 1^st^ segment of anterior cerebral artery, ICA: internal carotid artery, PCo: posterior communicating artery, PCA: posterior cerebral artery, P1: 1^st^ segment of posterior cerebral artery, SCA: superior cerebellar artery, BA: basilar artery.

The Circle of Willis (CoW) is an important anatomical structure in the vascular supply of the brain. In normal anatomy, the cerebral vasculature is comprised of three rooted trees: two corresponding to the left and right anterior circulation and one to the posterior circulation. The CoW is a network of arterial segments that provides a connection among these trees. The structure of a complete CoW and two variants are shown in Fig. 1. Physiologically, the arterial connections in the CoW are an important source of collateral blood flow and play an important role in pathologies such as stroke and mental illness^1^. The arterial segments of the CoW, however, are not present in all individuals. In an estimated 52% of individuals, one or more of the segments of the CoW are absent^1^. The role CoW variants play in stroke and related disease processes is a subject of active research.

An incomplete CoW is an independent predictor of stroke^2,3^. However, certain variants of the CoW may have a higher association with stroke in general^2,3^. In a study of 976 patients, the presence of an incomplete posterior CoW and incomplete anterior CoW was associated with a higher risk of stroke^3^. This study suggests that individuals with other particular variants may be more prone to stroke than individuals with a complete CoW.

On the other hand, a complete CoW is often used as surrogate for good collateral flow^4,5^. A complete CoW is associated with less hemorrhage when treated with tissue plasminogen activator^5^ and with increased probability of treatment targets on imaging beyond the conventional 4.5 hour treatment window^4,5^. Both results suggest that patients with a complete CoW may be treated more aggressively for stroke.

The analysis of the CoW and its variants can shed more light on the optimal management of stroke to better understand stroke risk factors. Moreover, consideration of vasculature is fundamental for diagnosis, treatment planning, surgery, and quantification of outcomes in many medical fields.

Typically, analysis of the CoW relies on expert interpretation and classification of radiological images. Though effective, reliance on human observers can be time consuming, expensive, and can introduce bias and variance into studies. Moreover, in a clinical setting, anatomical variants of the CoW are not always reported as, due to their frequency in the healthy population, such reporting would be burdensome to the radiological workflow. Supporting clinicians in evaluation of this anatomy via automatic or semi-automatic vessel segmentation and labeling could reduce the effort and financial burden while also minimizing bias and error.

Automatic classification of cerebrovasculature has been attempted previously using semi-automated approaches to segment and label anatomical structures. Bogunovic *et al*.^6^ present an automatic classifier of the arteries of the CoW in manually-segmented images. A Bayesian approach is used to train a model to label normal and variant anatomy using the angle of trajectory of each artery at their bifurcations and their relative position to each other as data (relational graph method). Using clinical data, Uchiyama *et al*. compare manually-segmented images of normal anatomy to a labeled atlas^7^. This comparison is done using registration, and feature correspondence between the sample image and atlas (Euclidean distance method). More recently, Kandil, *et al*. have used both a Bayesian framework and linear combination of discrete Gaussians^8^ and a 3D convolutional neural network^9^ to segment the cerebrovascular tree to estimate features for prediction of hypertension. These novel adaptive segmentation approaches to detect and quantify changes in the cerebrovasculature features extended beyond the CoW and demonstrate innovative feature extraction with clinical potential.

**Figure 2:**
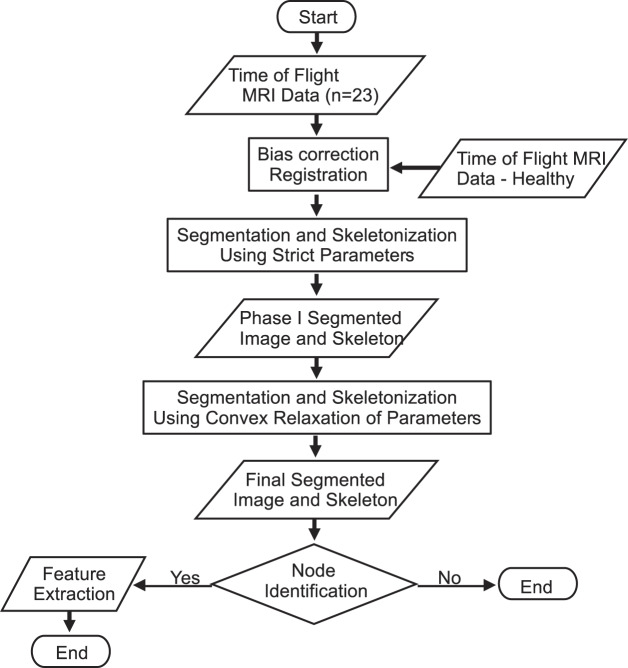
Outline of the image processing pipeline. The skeleton output from Phase 1 segmentation and skeletonization is preserved in Phase 2, allowing for increased feature extraction.

We present a method to automatically identify the branching points of the anterior circulation and bifurcation of the end basilar artery, i.e., the arterial supply to the CoW, and a method to identify patients with variant anatomy in the terminal branch of the internal carotid artery. The algorithm includes an image processing pipeline consisting of registration, segmentation, and skeletonization (a process that reduces a 3-D image to a one-voxel thick representation of its topological structure), and a post processing step that systematically compares the skeleton output to a pre-labeled atlas. The entire process is designed to work on clinically acquired data and is fully automatic. (Fig. 2)

Computer-aided radiological interpretation of magnetic resonance angiography images could dramatically reduce the cost of identifying CoW variants for research and allow for large scale data analysis. To this end, we present an approach that lays the groundwork for automatic classification of CoW variants.

## RESULTS

### Segmentation and skeletonization

**Figure 3:**
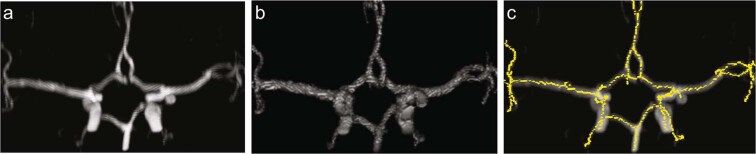
Output of segmentation and skeletonization pipeline on a participant with normal variant of the CoW. (**a**) Original image (**b**) Output after Phase 2 segmentation. (**c)** Output after Phase 2 skeletonization, which was used for post processing.

Iterations of segmentation and skeletonization (Phase 1 and Phase 2) were performed successfully on all data yielding the branching points of interest (intracranial ICA and basilar artery) as vertices (Fig. 3).

### End basilar branching

**Figure 4:**
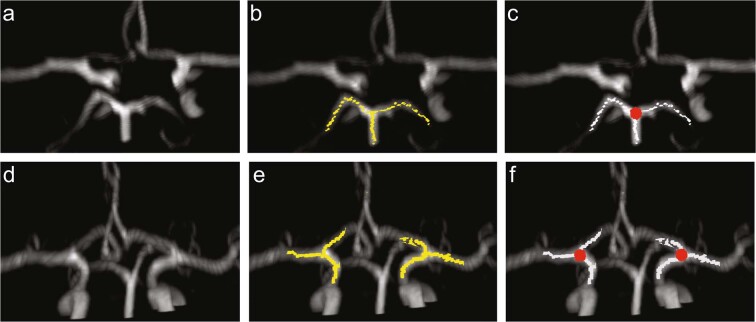
Identification of the posterior circulation (top row) and M1/A1 (bottom row) branching points. (**a**) Original image (**b**) Overlay of the original image with a skeleton representation of adjacent segments to the P1/P2/basilar artery branching point. (**c**) Identified node of posterior branching point (red circle) (**d**) Original image (**e**) Overlay of the original image with a skeleton representation of adjacent segments to the A1/M1 branching point (yellow), (**f**) Identified nodes of anterior branching point (red circles).

Identification of the “big nodes” of the end basilar circulation was successful in all 23 participants, with a typical result shown in the top row of Fig. 4. Panel 4b depicts the calculated skeleton in the region of the basilar artery branching point into the left and right P1 in yellow with the identified node indicated in red in panel 4c.

### End carotid branching

This initial implementation of the developed algorithm focused on identifying the presence of the M1/A1 branching point and, if two existed, determining if one of the A1 segments were variant, i.e., either hypoplastic or aplastic. Figure 4, bottom row, shows a typical output of the identification process for normal anatomy. Panel 4e depicts the calculated skeleton isolated to the region of M1/A1 branching point of the ICA (yellow). If this branching point was detected, the left and right nodes were identified as seen in panel 4 f (red circles).

**Figure 5:**
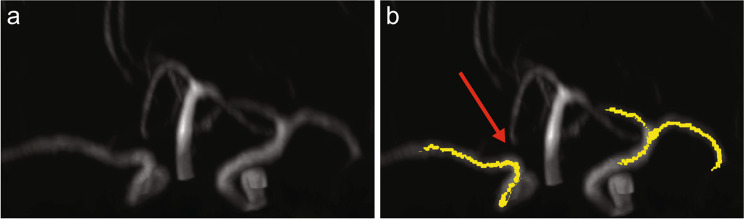
Successful detection of aplastic A1 segment. (**a**) Original image with the output of the image processing pipeline, (**b**) Overlay of the original image with skeleton (yellow) in which branching point of right ICA was not detected.

**Figure 6:**
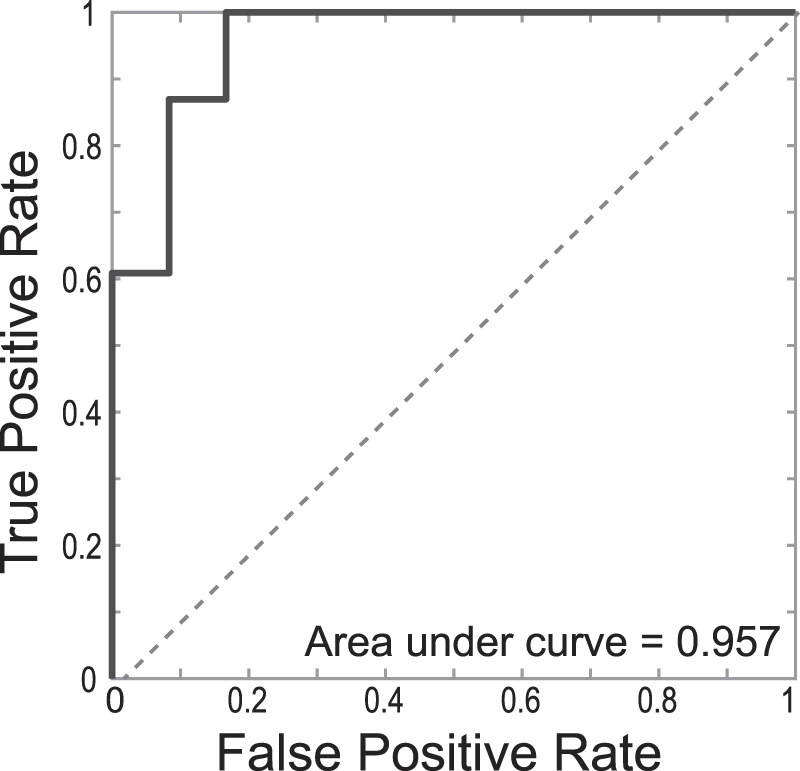
Receiver Operating Characteristic curve for $S$ to classify normal or A1 variant indicating an area under the curve of 0.957.

The M1/A1 branching points were correctly identified in all patients with normal CoW anatomy. In patients where the A1 anatomy was variant, the algorithm was designed to produce an error. Figure 5 shows such a case where the algorithm correctly identified A1 aplasia. The failure of the processing pipeline to identify the M1/A1 branching point was detected automatically using the score $S$ defined in Eq. (5). Figure 6 shows the Receiver Operating Characteristic curve for $S$ to classify normal or A1 variant with an area under the curve of 0.957. Receiver Operating Characteristic (ROC) curves show the tradeoffs that can be achieved by varying the threshold on S. An area under the ROC curve of 0.5 indicates chance classification while and area of 1.0 indicates perfect classification^10^. This illustrates the diagnostic ability of our developed processing pipeline for the sample tested.

Only hypoplastic variants were mislabeled as normal. To further identify these variants that were incorrectly labeled as normal, we examined the $F$ score. We derived a version of Eq. (1) using a linear best fit:

1 $${{\rm{F}}}_{i}^{\ast }=0.19367|{r}_{i}-{r}_{i-1}|-7.1199|{p}_{i}-{p}_{i-1}|$$

There were 23 patients with normal CoW anatomy and three with mislabeled variant anatomy. The mean ${F}^{\ast }$ score for patients with correctly labeled normal anatomy (n = 23) was −0.074 (SD = 0.161) and had a range from −0.330 to 0.230. The mean ${F}^{\ast }$ score for patients with variant anatomy (n = 3) incorrectly labeled as normal was 0.485 (SD = 0.174) with a range of 0.298 to 0.643.

## Discussion

### Segmentation and image processing

The presented processing pipeline was inspired by processing techniques for images of the lungs^11^. Tschirren *et al*. automatically labelled the human airway in computerized tomography images using image segmentation and skeletonization. The skeleton was then converted to a mathematical graph comprised of edges and nodes (i.e., branching points or vertices) where the edges represented airway segments and nodes represented the bifurcation points of these segments. The final mathematical graph was compared to a pre-labeled graph, derived from a population average, that served as an atlas.

We have built upon and modified the framework presented by Tschirren to address the inherent challenges of the cerebrovasculature. Though several methods exist for segmenting cerebral blood vessels^12–17^, no single method exists as a “state of the art” technique that could be applied to all images^18^. Additionally, unlike the lung, there are known variants of the CoW that prevent direct relationships between nodes and branching points. In fact, identifying these variants is one of the goals of the present work.

We acknowledge that more sophisticated methods do exist that could yield better segmentation results. However, we believe that the performance of any segmentation technique may be improved by using our multi-phase approach. Preserving the skeleton across many phases, allows extraction of key features in the image and overcomes heterogeneity in image acquisition protocols that are unavoidable in a clinical setting. We believe that the lack of a global segmentation method can be overcome by choosing segmentation parameters unique to each scanner.

In addition to challenges with heterogeneity, the time of flight magnetic resonance angiographies used in this study introduce challenges. The angle of flow in relation to the phase encode direction can significantly affect the contrast due to saturation effects. As a result, certain vessels, like the posterior communicating artery, are not visible in all images. Additionally, this hinders the voxel-level analysis of the contrast in the blood vessels via the score $F$. As a result, ither imaging modalities like computerized tomography angiography would not be affected by the angle of acquisition.

### Classification of nodes/A1 segments

Our work is distinguished from that of Tschirren^11^, Robben^17^, and Bogunovic^6^. In contrast to Bogunovic^6^ and Robben^17^, our work is based on images acquired in a clinical setting. Additionally, our goal was to label only key branching points and to do so without using any training data. Though Robben *et al*.^17^ use geometric properties of the anatomy, they do not rely on this for labeling. Our approach differs in that we use known anatomical features, such as wingspan, of each branching point for our labeling. As in Bogunovic^6^, these may include angles of arterial branching, but may include other features such as symmetry and distance relative to known landmarks. Again, we aim to use only these features and not any training data for labeling branching points. This approach was based on the idea that each of the elements in the CoW are anatomically unique. The scores used for classification here, namely $B$, $g$ and $S$, do not exhaust all the anatomically unique features of the branching points being studied. The presented work can serve as a cornerstone for refinement of these scores to yield better and faster classification.

Our work is similar to Uchiyama^7^ in the use clinical data, but differs in goals and approach. In addition to labeling major branching points, the present work also aims to classify anatomical variants of the A1 segment.

The objective of the developed post processing pipeline was to distinguish variant and normal A1 anatomy. Arithmetically, this entails determining if the M1/A1 anatomical identification process failed to detect the branching point based on score $S$. The parameters for the algorithm were empirically optimized for high sensitivity to aplasia in order to identify all aplastic A1 variants with additional sensitivity to A1 hypoplasia.

### Practical applications

The ability to automatically identify CoW variants and extract biomarkers from angiography images would significantly reduce financial and time burden on clinical personnel. Additionally, it would allow for large scale analysis as, once the segmentation parameters are chosen, the pipeline could process all of the images that are generated by a scanner.

Future work on this pipeline includes implementing more complex image processing techniques, generating alternative branching point classification techniques based on local anatomy of branching points, and working with images that have more uniform contrast. Other segmentation techniques like flux symmetry based active contouring^19^ or a stochastic approach designed for time of flight magnetic resonance angiography^14^ have the potential to yield better output for post processing. Such techniques may help resolve smaller segments of interest like the anterior communicating artery. These techniques may also yield better run times for the pipeline.

A central feature of our approach is using the local anatomy of key branching points, such as the end basilar circulation, for classification. Testing and implementing a more extensive library of scores, *e.g*., $S$, will improve performance and allow for more robust classification.

Another direction for future work may be decoupling the tasks of CoW variant identification and branching point identification and vessel segmentation. Knowing the CoW variant of a particular sample *a priori* simplifies the branching point identification task as the relationship between branching points is anatomically known. Given the CoW variant, our problem is indeed identical to that of Tschirren *et al*.^11^. One approach to this problem may be to train an entirely separate classifier to identify CoW variants. This may be done through artificial intelligence or traditional image processing of maximum intensity projections.

In addition to identifying variants, with further development, this approach has the potential to fully automate detection of certain biomarkers. For example, the developed pipeline could be implemented to identify variants in the CoW in studies of stroke risk as well as dementia. Since major arteries are labeled through our process, it may be possible to identify thinning of blood vessels and stenosis through intra-patient comparison as in Eq. (1). Additionally, since aneurysms do not change local topology of branching points, images with aneurysms would have a skeleton similar to one without aneurysms. Thus, such a classification process would not allow us to identify aneurysms through analysis of labeled branching points.

### Limitations

This study aimed to show the feasibility of a developed image processing pipeline. As such, the study was limited by its sample size and selection. The final step for classification of the hypoplastic variants that were mis-classified as “normal” is to apply a threshold to the $F$ score. Due to the limitations with the imaging modality (i.e., signal intensity heterogeneity within the vasculature) and small sample size, we did not attempt to optimize the classification threshold on the $F$ score. Nevertheless, out of the three variant samples that were mislabeled as normal, two resulted in $F$ scores that were significantly different than that of normal images. The $F$ classifier suggests that difference in minimum vessel radius is a good predictor for hypoplasia if and only if the average image contrast in the A1 segment is the same. This introduces sensitivity to hemodynamics and confounds the objective to assess anatomical structure. We believe that intra-patient comparisons of biomarkers, like average radius and voxel-based contrast, will likely yield a robust classifier with a larger sample size.

As the cohort included patients with indication for a head MRA, our sample may be biased or include subjects with pathologies that could potentially interfere with automated analysis. One such example includes a subject with a 10-mm right sided choroidal fissure cyst, which would appear as a hypodensity in the image, and potentially result in a mid-line shift. Nevertheless, the pipeline correctly identified the M1/A1 branching point, end basilar circulation, and appropriately classified this individual as having normal A1 anatomy. Additionally, the developed algorithm correctly classified branching points and variant status in a different subject with significant carotid artery disease.

Since the goal of the study was to assess feasibility, a small sample size was considered sufficient. However, this did not permit the training of a model for an intra-patient, comparison-based model for classification of A1 variants. A preliminary model is described by Eq. (1); however, more data are required to train a robust classifier.

The processing is initialized by raw images from the MRA and if these are low in signal to noise ratio, the brain extraction, bias correction, and segmentation have potential to fail. However, this is unlikely as the ToF MRA images are typically high SNR which is corroborated by the ability to implement methods such as nonlinear noise reduction (through FMRIB’s Software Library to average voxels with local voxels which have similar intensity to filter noise.

### Conclusion

We constructed a pipeline which generates an approximate segmentation and skeleton representation of the blood vessels of the brain, labels the M1/A1 bifurcation and the end basilar branching point in patients with complete CoW, and identifies individuals with variant A1 segments. Additionally, we demonstrated how intra-patient biomarkers may be generated and used to further classify hypoplastic A1 segments. We demonstrate the feasibility of using our approach to automatically classify important structures of the CoW and extract biomarkers from cerebrovasculature. With further development, we believe this approach may be used to automatically analyze clinically acquired images to generate biomarker data for research purposes or for clinical applications.

## Materials and Methods

There exist significant variants in cerebrovasculature, as seen in Fig. 1. We present an automatic image processing pipeline including pre-processing, two phases of image segmentation and skeletonization, and post-processing to result in a labelled image of the major anterior and posterior circulation feeding the CoW. Post-processing includes feature extraction and analysis. Figure 2 provides an overview of the image processing pipeline, which took on average 106.6 (±22.0) seconds.

### Patient population selection

This study was approved by an institutional review board with a waiver of consent due to limited risk for participants. All procedures followed were in accordance with institutional guidelines. Patients who underwent time of flight magnetic resonance angiography of the head between May 17, 2018 and January 6, 2019 at our academic medical center were eligible with 118 patients screened. Patients with evident or suspected vascular pathology or known variance in the posterior cerebral artery were excluded (n = 64). Additionally, all patients with scans affected by imaging artifacts, such as motion, were excluded. Eighty-two patients were excluded overall, 18 of whom had image artifacts. Of the 37 included patients, 23 had normal A1 anatomy and 13 had variant A1 anatomy. The median age of enrolled subjects was 61 years (interquartile range of 47–76), 31/37 (84%) were white, 3/37 (8%) black, and 3/37 (8%) Asian, 15/37 (41%) were Hispanic, and 24/37 (65%) were female. See Table 1 for subject demographics.

**Table 1:** Demographic information for included subjects.

	Included Patients (n = 37)
Age in years (st. dev.)	61 (19)
Sex (female)	65%
Race	
White	86%
African American	8%
Asian	8%
Ethnicity	
Hispanic	41%
Not reported or undetermined	59%
History of Stroke	19%
History of Hypertension	73%
Indication for Scan	
Suspected Stroke	70%
Suspected Hemorrhage	5%
Slurred Speech	5%
Other	20%

### Image acquisition

Images were acquired on a 3.0 T Siemens Skyra (Siemens Healthcare, Erlangen, Germany) using a 3D time of flight FLASH sequence with a repetition time of 21.0 ms, an echo time of 3.43 ms and flip angle of 18 $^\circ$. Axial slices were acquired with a field of view of 199 mm $\times$ 199  m $\times$ 40  m with 0.26 mm $\times$ 0.26 mm $\times$ 1.0 mm resolution.

#### Image processing: pre-processing

All image processing was performed in MATLAB 2019a (The Mathworks Natick, MA) and R (R Core Team, Vienna, Austria). Image pre-processing used the FMRIB Software Library (v6.0 Created by the Analysis Group, FMRIB, Oxford, UK) package for brain extraction^20^ and bias correction^21^. Images were then registered to a 0.5 mm $\times$ 0.5 mm $\times$ 0.5 mm atlas via the Advanced Normalization Tools^22^ implemented in R^23^. Subsequent histogram normalization via linear histogram matching method was performed using data from a healthy subject with normal CoW anatomy who was not analyzed in this study.

#### Image processing: segmentation and skeletonization

We aimed to capture major cerebral artery structure in the presence of heterogeneity in anatomy. The algorithm parameters were empirically chosen and informed by known anatomy of the CoW. The approach was iterative with two segmentation phases. This two-phase approach isolates “central” and “peripheral” features of the image. The segmentation outputs were related using skeletonization by preserving the skeleton generated by the strict segmentation during skeletonization of the relaxed segmentation step. By varying the rigidity of the segmentation steps, it is possible to generate regions of interest on the magnetic resonance angiography image that contain the major arteries of interest.

After pre-processing, images were segmented using adaptive K-means clustering and the Chan-Vese active contour algorithm^24^. The resulting segmented images were skeletonized using the Skeleton3D function^25,26^. This process was repeated using relaxed parameters for Phase 2 of the segmentation process. Adaptive clustering and each of the segmentation steps require pre-fixed parameters chosen empirically to allow for over-segmentation of the images. The minimum distance between two cluster centers was 10 pixels and minimum length of skeleton segment was 10 pixels with further fixed parameters discussed below.

#### Post processing: end basilar circulation

The normal anatomy of the basilar artery can be seen in Fig. 1. In many cases, the trajectory of the left-right Posterior Cerebral Artery (PCA) and the left/right superior cerebellar artery (SCA) is such that they are represented as branching point with 5 branches, the fifth being the basilar artery itself. Post segmentation, the trajectory of the PCA is visible in all images, whereas that of SCA is not. However, since only the PCA plays a role in the CoW, the correct labeling of the SCA would not influence classification of CoW variants. Thus, instead of looking for a single (or triple) voxel representation for a vertex, we looked for “big nodes,” or a region in the image, where all the visible arteries associated with this branching point would meet. Big nodes are defined as a region up to 5 mm in radius. This included the left and right PCA and the basilar artery in all images and one or both SCA in some.

A similar approach to the anterior circulation was taken with the following steps:

- Tree candidate “big nodes” were identified according to the anatomy to be within 30 mm of the corresponding node in the atlas.
- The center of mass and position relative to the median sagittal plane were obtained and each node was given a score by:

2 $$B(n)={d}_{y}(n,m)-h{(n)}_{x},$$

where the function $h(n{)}_{x}$ computes the projection of the center of mass of all branches in the $x$ direction.

- The big node with the highest $B$ score is assigned as the branching point of the basilar artery.

#### Post processing: end carotid circulation

There are many anatomical structures near the M1/A1 branching point of the intracranial internal carotid artery (ICA) including the posterior communicating artery, the anterior cerebral artery, and the ophthalmic artery (and the structure of the ICA itself). To identify the M1/A1 bifurcation, we used geometric and physical properties specific to this branching point. The analysis was done on left and right side separately. The steps taken were as follows:

- identification of the three candidate M1/A1 branching points as those near each of the left and right M1/A1 locations in the atlas,
- generating the projection of the edges and nodes associated each candidate branching point onto the ‘y’ axis (anatomically referring to the left to right axis),
- generating a score for a pair of nodes *(i,j)* defined as:

3 $$M(i,j)=\alpha |{e}_{i}-{e}_{j}|-g(i,j),$$

where ${e}_{i}$ is the absolute value of maximum ‘y’ projection of the branches from the node ‘i’ (capped at 15 mm) and $g(i,j)$ is defined as:

4 $$g(i,j)=\beta d(i,j)-max({d}_{y}(i,m),\,{d}_{y}(i,m)),$$

where $d$, ${d}_{y}$ are the 3-D Euclidean distance and y-projected Euclidean distance respectively and $M$ is the y-projection of the medial sagittal plane. The constants α and β are scaling parameters that were chosen manually.

- The node with the highest $M$ score derived from Eq. (3) is labeled as the M1/A1 branching point.

Once the highest score nodes were identified, the nodes were given a score based on their symmetric properties and the processing transitions to a symmetric evaluation. This symmetry score is given as follows:

5 $$S(i,j)=g(i,j)-\frac{|{y}_{i}-{y}_{j}|}{min{({y}_{i},{y}_{j})}^{2}},$$

where ${y}_{i}$ are the absolute value of y-projections of the node offset by the median sagittal plane.

The choice of scores in Eqs. (3) and (5) are complementary. $M$ penalizes the selection of nodes that do not meet the geometric properties of the M1/A1 branching point, such as its projections in the y-direction and symmetry, and in Eq. (5), $S$ penalizes an asymmetric distribution of nodes near the median sagittal plane. Due to the geometric properties of the M1 and A1 segment, in the absence of an A1 segments, $S$ prefers the selection of node pairs that are close to the median sagittal plane. This selection is then penalized by $S$. The square in the denominator can be justified by first having to normalize the difference ${y}_{i}-{y}_{j}$ and then penalizing an asymmetric selection.

#### Post processing: A1 variant analysis

The analysis of A1 segments aims to classify patients into two categories: variant versus normal. Variant anatomy may be further classified into aplastic and hypoplastic, where aplastic refers to complete absence of the A1 segment and hypoplastic refers to significant diminution of one A1 segment relative to the isolateral A2 segment and the contralateral A1 segment. The algorithmic classification of variant anatomy was done solely based on the M1/A1 identification pipeline. Failure of the pipeline to identify a node indicates presence of variant anatomy. The gold standard for algorithm validation was expert classification (variant/normal) by a certified Neurologist with over 27 years-experience (SJW).

#### Post processing: feature extraction

In cases where the A1 segment is identified, the segments were given the following score:

6 $${F}_{i}=a|{r}_{i}-{r}_{i-1}|+b|{p}_{i}-{p}_{i-1}|+H(i,i-1)-c|{T}_{i}+{T}_{i-1}|,$$

where $i$ and $i-1$ are the left and right A1 segments (or vice versa), ${r}$ is the average vessel radius, ${{p}}_{i}$ is the average signal intensity, $T$ is the vessel tortuosity, and $H(i,i-1)$ is an asymmetric function that is 1 if only $i$ has an end point and 0 otherwise.

The physical properties of the vasculature identified in Eq. (1) were computed using an approximation of the A1 segment derived from the output of Phase 2 segmentation and skeletonization. The volume that represents the artery was taken to be the union of all the spheres, centered at the skeleton, that are within the final segmentation output. $r$ was approximated as the average radii of these spheres and $p$ was approximated as the average voxel value. The function $H(i,i-1)$ was computed using the skeleton itself and lastly, $T$ was approximated by the length of the skeleton representation of the A1 segment and the distance between its end points.

This linear model in Eq. (1) can be used as a classifier to further identify variant anatomy in cases where the image was classified as “normal” based on $S$. Among the patients where the M1/A1 node was correctly identified, three patients with variant anatomy were mislabeled as normal. Using these three patients and the correctly labeled normal patients (n = 23), linear regression was used to train a model, based on $F$, to predict normal ($F=0$) or variant ($F=1$). A threshold for the fitted score was manually chosen for classification. Variables with insignificant p-values in Eq. (1) were disregarded.

### Research involving human participants and/or animals

The studies presented here have been approved by the University of Texas at Austin Dell Medical School Institutional Review Board (study number 2018-02-0044) and have been performed in accordance with the ethical standards as laid down in the 1964 Declaration of Helsinki and its later amendments or comparable ethical standards.

### Informed consent

Informed consent was waived by the University of Texas at Austin Institutional Review Board for this study. This retrospective study was approved under the study number 2018-02-0044.

## Data Availability

The datasets generated during and/or evaluated during the current study are available from the corresponding author on reasonable request.
